# Colchicine causes prenatal cell toxicity and increases tetraploid risk

**DOI:** 10.1186/s40360-019-0365-z

**Published:** 2019-11-13

**Authors:** Ding Wang, Yingjun Xie, Minyi Yan, Qianying Pan, Yi Liang, Xiaofang Sun

**Affiliations:** Key Laboratory for Major Obstetric Diseases of Guangdong Province, Key Laboratory of Reproduction and Genetics of Guangdong Higher Education Institutes, Experimental Department of Institute of Gynecology and Obstetrics, The Third Affiliated Hospital of Guangzhou Medical University, No. 63 of Duobao Road, Guangzhou, 510150 Guangdong China

**Keywords:** Colchicine, Chorionic villus cells, Amniotic fluid cells, Cell cycle arrest, Tetraploid generation

## Abstract

**Background:**

Colchicine is a clinical medicine used for relief from gout and familial Mediterranean fever. Because of its toxic effects, intravenous injection of colchicine has been banned, but it is still widely administered orally. We assayed the toxic effects of colchicine in cultured primary chorionic villus cells and amniotic fluid cells to interpret its influence on the placenta and foetus.

**Methods:**

Bright field record and cell count kit 8 were used to value cell viability. Flow cytometer was used to identify cells markers, cell cycle and cell apoptosis. G-banding was used for karyotype analysis for sample genetic and drug effect evaluation.

**Results:**

Chorionic villus cells and amniotic fluid cells were characterized as mesenchymal cells that share most cell surface markers and have a similar response to colchicine. Colchicine did not induce a decline in cell viability at low concentrations but suppressed cell proliferation by arresting the cell cycle in the G2/M phase and increased the risk of tetraploid generation in a small subset of cases.

**Conclusions:**

Our study revealed the results of a colchicine-induced toxicity test in prenatal cells and determined the anti-mitotic biologically functional dose and manner of administration that might reduce the risk of tetraploid generation.

## Background

Colchicine is a natural product extracted from the autumn crocus (*Colchicum autumnale*, the name is derives from the Greek word) plant, which is a taxonomic species within the family Colchicaceae. Owing to its biological activity, colchicine has been used in medical care for centuries. There are two main medical uses of colchicine: first, colchicine was regarded as an anti-inflammatory agent used in treating familial Mediterranean fever (FMF), Behcet’s disease and gout; second, colchicine was used as a cardioprotective agent to treat acute and recurrent pericarditis and other cardiovascular-associated abnormalities [1]. In 2009, the United States Food and Drug Administration (FDA) approved colchicine for the treatment of gout and FMF. Based on its pharmacological effects, some clinical trials are in progress and, for some, the results have recently been published, such as those on stroke prevention [2] and enhanced graft survival [3], creating new means for treating disease and promoting wide colchicine medicinal usage. Colchicine is one of the oldest drugs still used today.

Side effects of colchicine accompany its medical usage. At recommended doses, 10% of the patients show some gastrointestinal distress syndromes, such as nausea, vomiting, and particularly diarrhoea. With overdose, it is highly toxic, even fatal [4]. Considering the side effects, the FDA withdrew approval for intravenous colchicine and provided guidelines for reducing the recommended dose and for concomitant use of CYP3A4 and P-glycoprotein inhibitors [5], which target the toxic effects caused by pharmacological use of colchicine.

Most toxicological research on colchicine focuses on treating patient but seldomly focuses on pregnant women or their foetuses. Chorionic villus tissue is a constituent of the placenta, and chorionic villus cells (CVCs) are mesenchymal cells derived from the chorionic villus, from which biopsy samples are taken at the end of the first trimester. Amniotic fluid contains foetal membrane cells and foetal cells, and amniotic fluid cells (AFCs) are mesenchymal cells derived from the amniotic fluid and are extracted during the second trimester. Primary cultures of CVCs and AFCs to obtain chromosomes for G-banding are widely used in prenatal genetic diagnoses. CVCs and AFCs also have with the same biochemical and genetic characteristics as the placenta and foetus, which are in contact with the amniotic fluid. For this study, we used cultured primary chorionic villus cells (CVCs) and amniotic fluid cells (AFCs) to evaluate the toxic effects of colchicine on the placenta and foetus.

## Methods

### Specimens

This study was approved by the Ethics Committee of The Third Affiliated Hospital of Guangzhou Medical University (Approval number: 2017–001). Six pregnant patients signed informed consent and were told that the diagnostic sample would be used for research. Four pregnant women at 18–21 weeks gestation were undergoing amniocentesis, and two at 12 weeks gestation were undergoing chorionic villus sampling. After the cytogenetic diagnoses were completed, the four AFC lines and two CVC lines were used in this study.

### Cell culture

The clinical samples were 8 ml amniotic fluid or 100 g chorionic villus tissue for each patient. For primary culture, amniotic fluid was centrifuged in 300G and chorionic villus tissue was cut into approximately 1 mm^2^ tissue masses. Then, cell precipitates (or the tissue masses) were resuspended in 4 ml amnioMAX-C100 medium (Cat#12558011, Invitrogen Life Technologies, Carlsbad, CA, USA) and seeded into two 25-cm^2^ culture flasks where they were maintained for 7–9 days at 37 °C in 5% CO2. Cells were detached by 0.05% trypsin (Cat#25300054, GIBCO, Invitrogen China Limited, Shanghai, China) for subculture. Clinical cytogenetic diagnosis was performed on passage 1. AFCs and CVCs were subcultured in complete MEM medium (Cat#12571071, GIBCO) supplemented with 20% foetal calf serum (Cat#10100, GIBCO) and 100 U/ml of penicillin and streptomycin (Cat#10378016, Invitrogen). Cells from passages 3–5 were used for the toxicity assays of colchicine (Cat# S2284, Selleck Chemicals, Houston, TX, USA). Colchicine was dissolved in MEM medium, for dose-dependent, cells were treated by 0, 0.15, 0.3, 0.6, 1.2 and 2.4 μg/ml colchicine for 3 h; for time-dependent, cells were treated by 0.15 μg/ml colchicine for 0, 12, 24, 48 and 72 h. As colchicine diluted in MEM, untreated group was control.

### G-banding analysis

The P1 subcultured cells were treated with 0.15 μg/ml colchicine for 3 h, detached by 0.05% trypsin, subjected to hypotonic treatment with 0.02% sodium citrate and 2% potassium chloride and fixed in a mixture of methanol and acetic acid in 3:1 (volume to volume). The fix cells were dripped on cold slides and stained with Giemsa for metaphase chromosome analysis that was conducted with an Ikaros system (Carl Zeiss AG, Oberkochen, Germany).

### Cell viability assay

A total of 5000 cells were counted in each well of 96-well plates, with triplicate sets for each group. After treatment with different drug concentrations at specific time points, the cell viability was assessed using a commercial CCK-8 (Cell Counting Kit-8) assay (Cat#C0039, Beyotime Biotechnology, Shanghai, China).

### Cell surface marker assays

The cells were detached by trypsin and stained by the following fluorescence-conjugated antibodies: CD14-APC, CD34-PE, CD45-FITC, CD105-FITC, CD29-PE, CD44-FITC and CD73-APC. The population analysis was conducted on an Attune NxT flow cytometer (Invitrogen).

### Cell cycle assay

Drug-treated cells were detached by trypsin and fixed in 70% ethanol overnight. The fixed cells were assayed using a cell cycle and apoptosis analysis kit (Cat#C1052, Beyotime Biotechnology) on a flow cytometer. The fluorescence intensity was used to distinguish different phases of the cell cycle.

### Cell apoptosis assay

1 × 10^6^ cells were seeded in to a 100 cm^2^ dish overnight for attachment. Drug treatment was accessed by change medium supplemented with 0.15 μg/ml colchicine. After drug treatment, the cells were stained with an annexin V-FITC apoptosis detection kit (Cat#C1062M, Beyotime Biotechnology) and counted by a flow cytometer for analysis. The cells in early apoptosis were the annexin V-positive and PI (propidium iodide)-negative cells, and the cells in late apoptosis were the annexin V- and PI-positive cells.

### Statistical analysis

The cell viability was expressed as the mean ± SD, and the percentage of the total number of cells in metaphase was expressed in a scatter diagram. The number of diploid and tetraploid cells in each analysis are presented in the Table 1. For two groups, the data were statistically analysed by paired Student’s t-tests; for different concentrations of colchicine and different time points, the data were statistically analysed by ANOVA, and a *p*-value of < 0.05 was considered statistically significant. A chi-square test was used to compare the proportion of tetraploid cells in the control group and in the drug-treated groups during each metaphase analysis. A p-value of < 0.05 was considered statistically significant for either group.

**Table 1:** Statistical analysis of diploid and tetraploid cells in the prenatal samples

	Karyotype	Tetraploid VS. Diploid		
		Control	0.075 μM Colchicine (*P* Value)	0.15 μM Colchicine (*P* Value)
AFC 1	46, XY	2 VS. 20	0 VS. 15 (>0.05)	0 VS. 19 (>0.05)
AFC 2	46, XX	10 VS. 89	3 VS. 49 (>0.05)	8 VS. 65 (>0.05)
AFC 3	46, XX	0 VS. 15	4 VS. 22 (>0.05)	2 VS. 25 (>0.05)
AFC 4	46, XY	2 VS. 25	3 VS. 29 (>0.05)	**10 VS. 19 (0.014)**
CVC 1	46, XY	1 VS. 78	3 VS. 87 (>0.05)	0 VS. 90 (>0.05)
CVC 2	46, XX	3 VS. 80	5 VS. 97 (>0.05)	2 VS. 61 (>0.05)

Significant data are in bold

## Results

### Isolation, culture and characteristics of prenatal cells

**Fig. 1:**
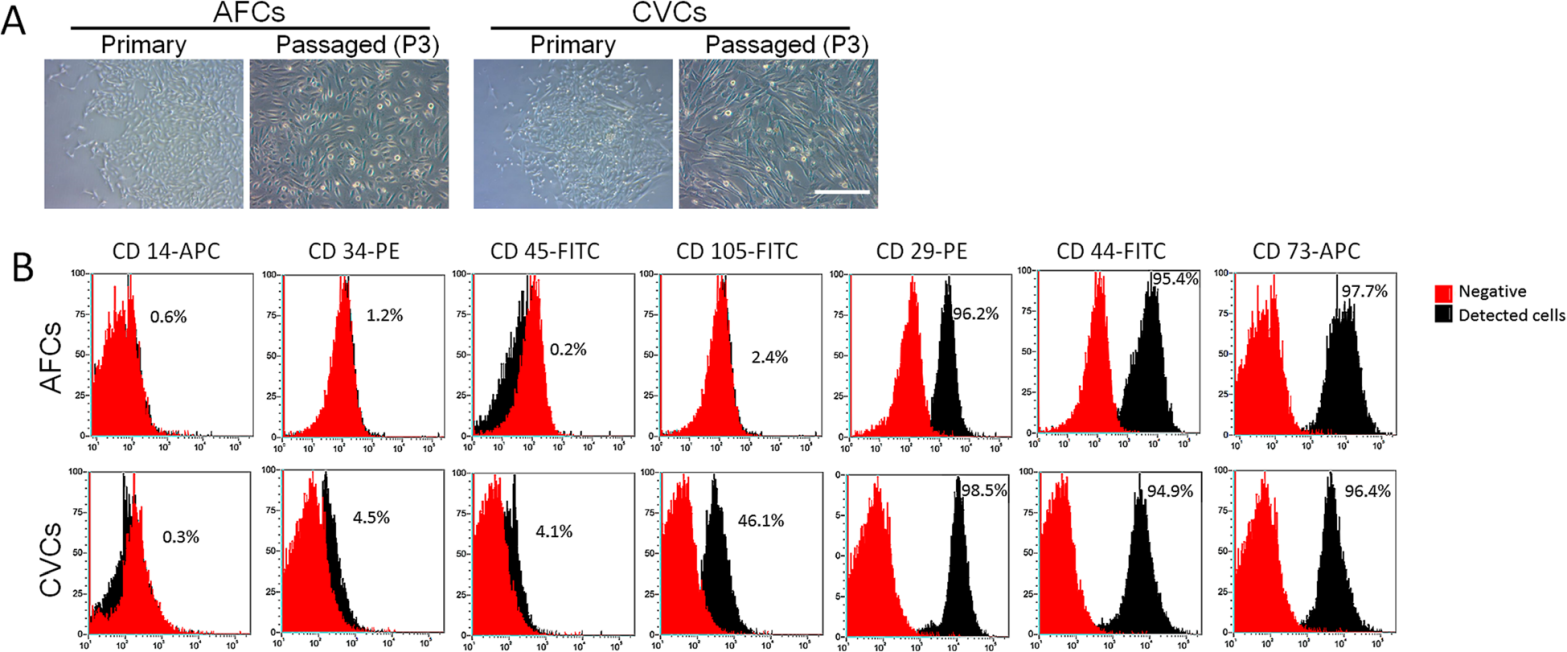
The isolation and characteristics of AFCs and CVCs. **a** The AFCs and CVCs in primary culture and subculture are indicated. **b** The surface markers of the subcultured AFCs and CVCs are indicated. The peak area in red represent negative markers, and the black represents markers detected in the cells. The number in the plot indicates the ratio of each positive marker

After the initial culture, cells (amniotic fluid) and tissues (chorionic villus) were maintained in culture medium for 7 days, and spindle-shaped fibroblast-like cells appeared. Then, the cells were fed fresh medium for 3 days during their expansion, and they formed primary colonies with unclear edges (Fig. 1a). The colonies were detached into single cells by trypsin and re-seeded into the culture flask for the subculture. The morphology of the CVCs and AFCs was homogeneous after three generations of subculture (Fig. 1a). The cell surface markers were identified by flow cytometry and then used for the colchicine-induced toxicity study. The CVCs and AFCs were identified as one kind of mesenchymal cell with shared markers: they were positive for CD29, CD44 and CD73 and were negative for CD14, CD34 and CD45, but the CVCs and AFCs had different levels of CD105 expression (Fig. 1b).

### Colchicine affects cell viability in a time- and dose-dependent manner

**Fig. 2:**
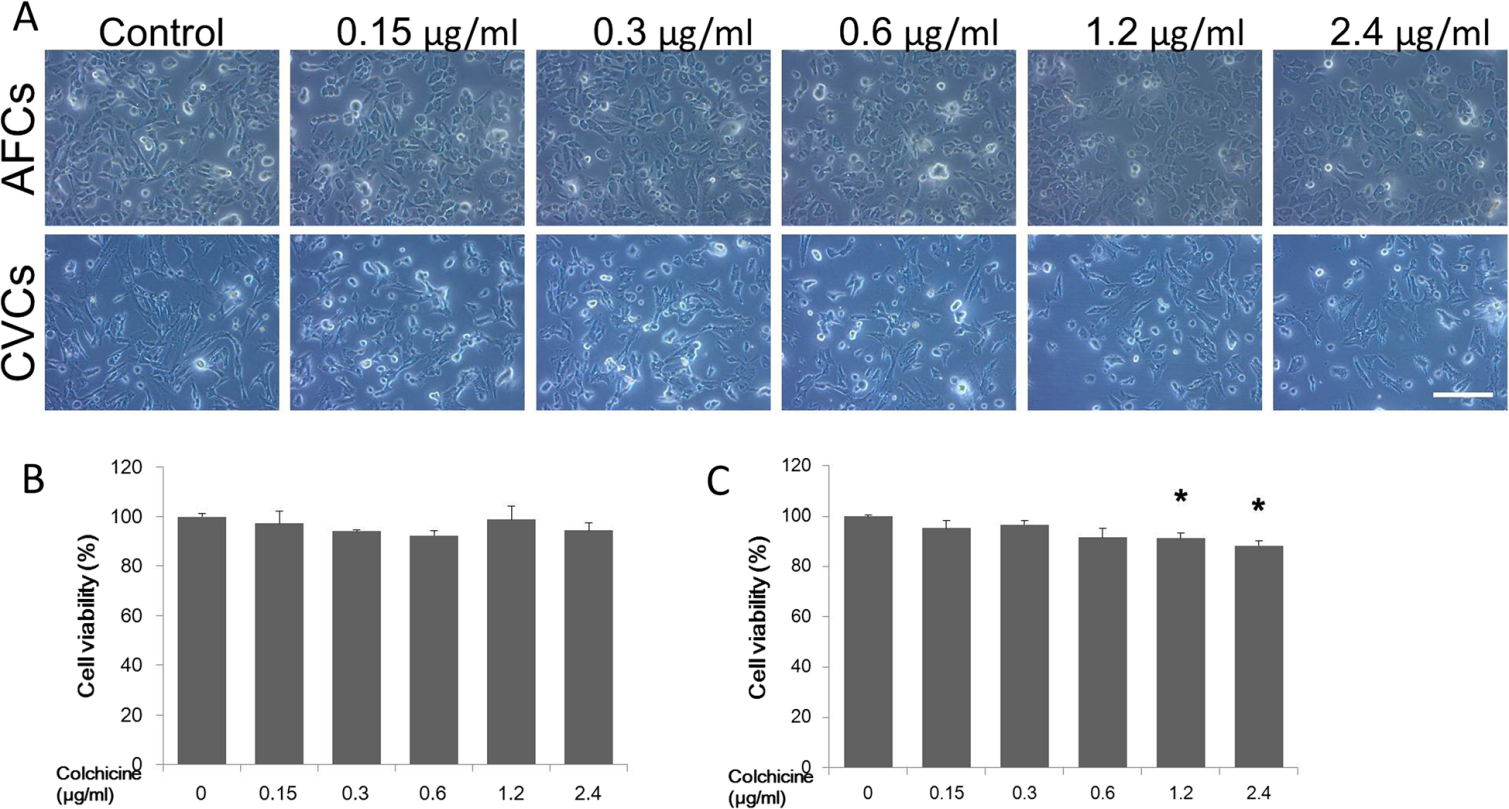
The dose-dependence of colchicine-induced toxicity in the AFCs and CVCs. The cell morphology (**a**) and cell viability (**b** for AFCs and **c** for CVCs) indicated for the AFCs and CVCs treated with different doses of colchicine for 3 h. (*n* = 3, *P < 0.05 versus untreated group)

**Fig. 3:**
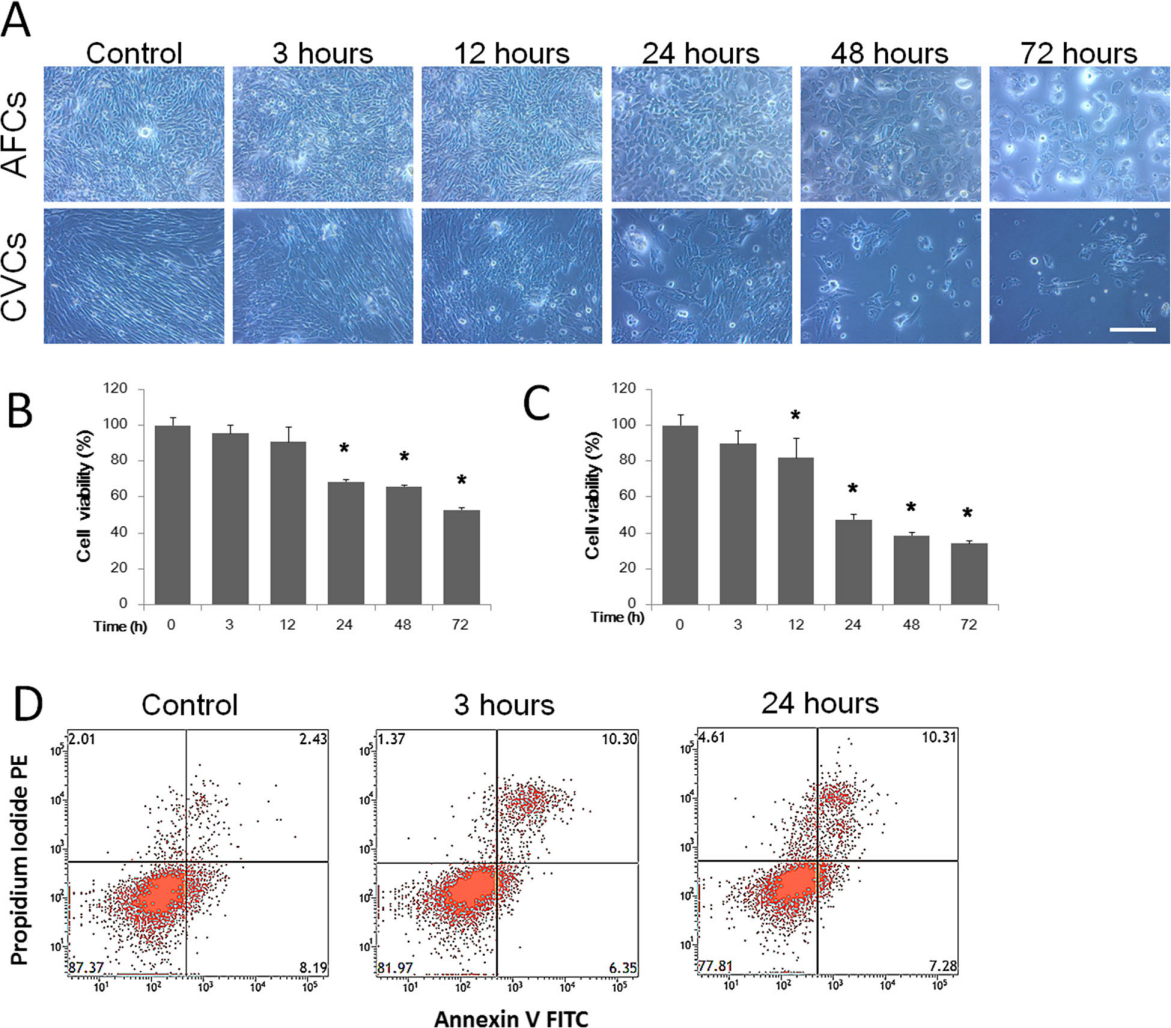
The time-dependence of colchicine-induced toxicity in AFCs and CVCs. The cell morphology (**a**), cell viability (**b** for AFCs and **c** for CVCs) and annexin V and PI ratio (**d**) are indicated for AFCs and CVCs treated at different times with 0.15 μg/ml of colchicine. (*n* = 3, *P < 0.05 versus untreated group)

To evaluate the colchicine-induced toxicity in prenatal cells, we recorded the cell morphology and conducted cell viability analysis. The CCK-8 assay was used for the cell viability evaluation. The CVCs and AFCs displayed different sensitivity of colchicine, with the CVCs more easily induced by colchicine treatment to undergo cell death than the AFCs. For the dose-dependence test, the prenatal cells were treated for 3 h, and there were no significant changes in AFC morphology or cell viability with increasing concentrations of colchicine (from 0 to 2.4 μg/ml), while 1.2 μg/ml and 2.4 μg/ml of colchicine induced significant decline in CVC viability (Fig. 2a, b and c). For the time-dependence test, the prenatal cells were treated with 0.15 μg/ml colchicine, and a significant decline in cell viability was found for both AFCs (after 24 h) and CVCs (after 12 h) (Fig. 3a, b and c). Furthermore, we used flow cytometry to determine the colchicine-induced cell death ratio of the AFCs, as determined by cell viability. Although the cell viability did not change after a 3 h treatment with 0.15 μg/ml colchicine, there was a significant increase in the ratio of double-positive annexin V and propidium iodide (PI) cells compared with the control group. However, there was no significant change between 3 h and 24 h of treatment (Fig. 3d).

### Colchicine reduces cell proliferation ability

**Fig. 4:**
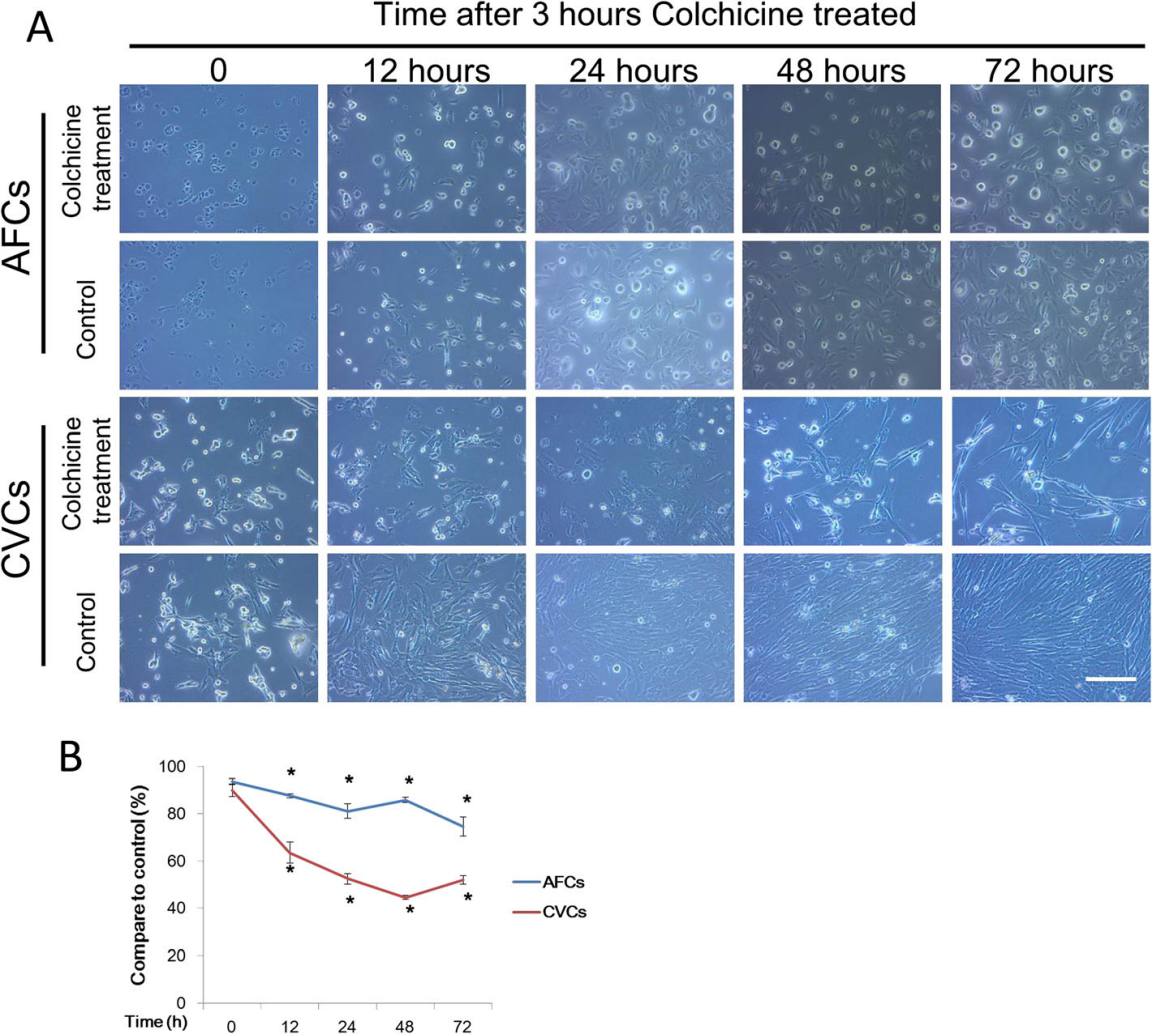
The cell proliferation assay after colchicine treatment in AFCs and CVCs. The results for the cell morphology (**a**) and cell viability (**b**) assays at different times are indicated for AFCs and CVCs treated with 0.15 μg/ml of colchicine for 3 h (*n* = 3, *P < 0.05 versus untreated group)

Although there was no viability change in AFCs and CVCs after 3 h of 0.15 μg/ml colchicine treatment, to determine the comprehensive effect of the treatment, we cultured the prenatal cells without colchicine for 72 h and assayed cell viability at different time points to determine whether colchicine treatment affected cell proliferation. Compared to the control group, there were no significant differences in the colchicine treatment group at the initial point in terms of cell morphology or cell viability (Fig. 4a and b). The colchicine treatment group did not show cell proliferation following culture, while the control group grew over 72 h in culture (Fig. 4a). This result prompted us to conclude that colchicine treatment suppressed the cell proliferation ability compared with the control group, as determined by cell viability assay after 12 h in culture (Fig. 4b).

### Colchicine causes cell cycle arrest and induces polyploids

**Fig. 5:**
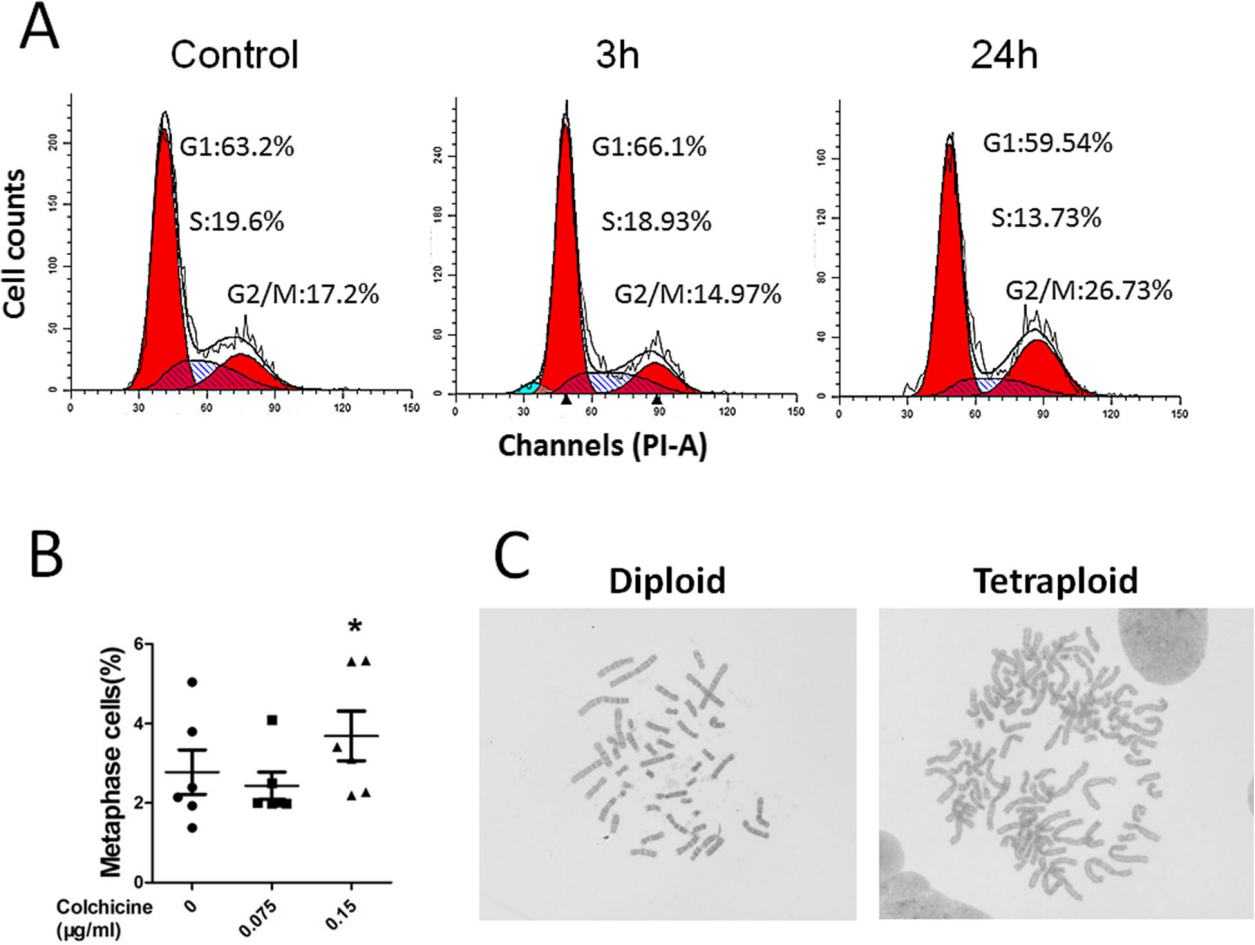
Colchicine arrested the cell cycle in prenatal cells (**a**) The cell cycle of prenatal cells treated with 0.15 μg/ml colchicine are indicated for different times. **b** The number of metaphase cells to total prenatal cells is indicated after treatment with different concentrations of colchicine for 3 h (AFCs, *n* = 4 and CVCs, *n* = 2. *P < 0.05 versus untreated group). **c** Indicated diploid and tetraploid in prenatal cells

One biological function of colchicine is its inhibition on spindle formation during mitosis; therefore, colchicine was used to treat prenatal cells for cytogenetic diagnosis at a concentration of 0.15 μg/ml for 3 h. We studied the influence of colchicine on mitosis and cytogenetics by cell cycle analysis and G-banding karyotype analysis. The cell cycle distribution was assessed at 3 h and 24 h after 0.15 μg/ml colchicine treatment. There was no change after 3 h, while the number of G2/M cells increased after 24 h (Fig. 5a). The G-banding karyotype analysis was performed in 3 h with 0.15 μg/ml and 0.075 μg/ml colchicine treatment. For the metaphase cell ratio, no differences were found for the 0.075 μg/ml treatment, while the number of cells treated with 0.15 μg/ml increased compared to the number of untreated cells (Fig. 5b). Most of the cells in metaphase were diploid, but there were tetraploid cells in most cell groups (Fig. 5c). By statistical analysis, we determined that there were no differences in the colchicine treatment group compared to the control group, while a significant increase in tetraploid cells was found in one of the AFC groups treated with 0.15 μg/ml colchicine (Table 1).

## Discussion

Pregnant women exhibit specific and substantially different anatomical and physiological characteristics, resulting in the necessity to evaluate drug pharmacokinetics, functional mechanisms and toxic effects comprehensively. On one hand, the drug must benefit the individual; on the other side, attention must be paid to minimize any side effects not only in the mother but also in the foetus [6]. Colchicine is a clinical medicine that has been in continuous use, and epidemiological studies have suggested that clinical colchicine usage is not associated with foetal malformations or miscarriage, but its anti-mitotic properties may cause pre-term birth, short gestational age and low birthweight [7]. In this study, we used primary prenatal cells to evaluate the cell toxicity induced by colchicine and to uncover the cell biology of the colchicine-induced toxicity in the placenta and foetus.

The CVC and AFC models can be used to evaluate drug cell toxicity to the placenta and foetus. Primary cultures of CVCs and AFCs are widely used in prenatal diagnosis, especially as the foundation of cytogenetic determination. The AFCs and CVCs were harvested at different gestational ages and represent different tissues. Chorionic villus tissue was acquired by biopsy of the placenta at 11–12 weeks of gestation, which was an important period of its growth and maturity [8]. The CVCs were acquired from and represent the placenta. Amniotic fluid was extracted at 18–21 weeks gestation, at which time, the primary circulatory system of the foetus is established [9]. The amniotic fluid is the urine of the foetus, being mainly composed of water but also containing foetal faeces and exfoliated cells of the body and amniotic membranes. The acquired AFCs were regarded as foetal cells. In clinical diagnosis, the CVC genetic report reflects the placenta, and analysis of the AFCs was used to diagnose the foetus, especially for mosaicisms [10]. We used clinically obtained CVCs and AFCs to test colchicine-induced toxicity, partly to determine the influence on the placenta and foetus body. The morphology of passaged CVCs and AFCs differed, with CVCs being slender with clear boundaries, and the AFCs being flat; both were homogeneous as determined by bright field observation. Actually, CVCs [11] and AFCs [12] were mesenchymal stromal cells (MSCs), which represent a part bio-function of their sources. Here, we check the surface biomarkers of MSCs [13]. The AFCs shared most of their cell surface markers with the CVCs: both were positive for CD29, CD44 and CD73, and both were negative for CD14, CD34 and CD45; however, CD105 was expressed differently in the two cell lines, and long-term culture increased the CD105 ratio [14]. In our study, although AFCs and CVCs showed the same trend in terms of induced cell toxicity, the CVCs were more sensitive to colchicine in terms of cell viability and proliferation, showing more changes than AFCs, which suggests that colchicine affects the placenta much more than the foetus body, findings that are consistent with the epidemiology studies [7].

To precisely interpret the colchicine-induced toxicity of the placenta and foetus, we need a complex biological model and the ability to monitor the system. The micro-environment is complex and includes multiple types of cells and a complicated extracellular matrix that changes quickly, a situation quite different from our cell model. On the other hand, the concentration of colchicine in our study was higher than the peak plasma concentrations [15]; however, despite the low concentrations, the effect over time might be greater, as the exposure to colchicine would be maintained throughout the entire pregnancy.

It is necessary to evaluate colchicine toxicity in the placenta and foetus. Colchicine is the first-line therapy for gout and FMF due to its anti-inflammatory properties [16]. Three proteins preferentially bind to colchicine: tubulin, CYP3A4 and P-glycoprotein. The anti-mitotic properties of colchicine that accelerate cells proliferation derive from tubulin binding, the metabolism of colchicine depends on CYP3A4 and P-glycoprotein in the liver, and colchicine is excreted via P-glycoprotein in the kidney [17]. Pregnant women lack an adequate response to colchicine because of an increase in hepatic CYP3A4 enzyme activity [18]. The increase in the dose administered to pregnant women could result in side effects in the foetus, as placenta cells express P-glycoprotein as an important transporter for substance exchange between the mother and foetus [19].

Our prenatal cell toxicity data may reflect the influence of colchicine on the placenta and foetus, but it did not completely mimic the toxic reaction in vivo. For clinical cytogenetic diagnosis, 0.15 μg/ml of colchicine was used to arrest rapidly proliferating cells in metaphase [20]. In our study, prenatal cell viability was not affected in 12 h by 0.15 μg/ml colchicine treatment, but the ratio of late apoptotic cells (annexin V- and PI-double positive cells) was significantly increased; the continuous treatment over 24 h resulted in a decline in cell viability but not an increase in apoptotic cells. Furthermore, treatment with 0.15 μg/ml of colchicine for 3 h did not affect cell viability but limited cell proliferation in the continuous cell culture. Another biological phenomenon of colchicine resulted in an increase in the ratio of mitotic tetraploid cells, which is common in plants [21]. Although the total number of tetraploids was low and they were not found in all the samples, it is necessary to take notice of the risk for developing genome abnormalities. Based on these findings, we conclude that the biological function of colchicine was arrest of the cell cycle but not induction of cell death.

## Conclusion

In summary, we used primary cells derived from chorionic villus and amniotic fluid to assay colchicine-induced toxicity. Colchicine did not induce a decline in cell viability at low concentrations, but chronic exposure triggered suppressed cell proliferation by arresting the cell cycle in the G2/M phase and increased the risk of tetraploid generation. Our study reflects the influence of colchicine-induced toxicity in the placenta and foetus, but additional studies are needed on a more representative biological model that can be precisely monitored for interpretation of the relevant biological mechanisms.

## Data Availability

All data generated or analyzed during this study are included in this published article (and its supplementary information files).

## Abbreviations

AFCs: Amniotic fluid cells
CCK-8: Cell Counting Kit-8
CD: Cluster of Differentiation
CVCs: Chorionic villus cells
CYP: Cytochrome P450 proteins
FDA: Food and Drug Administration
FMF: familial Mediterranean fever
PI: Propidium iodide
